# Structure Optimization of Neuraminidase Inhibitors as Potential Anti-Influenza (H1N1Inhibitors) Agents Using QSAR and Molecular Docking Studies

**Published:** 2014

**Authors:** Poonam Inamdar, Shashikant Bhandari, Bhagyashri Sonawane, Asha Hole, Chintamani Jadhav

**Affiliations:** *Department of Pharmaceutical Chemistry- PG Wing, AISSMS College of Pharmacy, Pune-01, Maharashtra, India. *

**Keywords:** Anti-influenza agents, Drug design, Neuraminidase Inhibitors, Molecular Modelling, Optimization

## Abstract

The urgent need of neuraminidase inhibitors (NI) has provided an impetus for understanding the structure requisite at molecular level. Our search for selective inhibitors of neuraminidase has led to the identification of pharmacophoric requirements at various positions around acyl thiourea pharmacophore. The main objective of present study is to develop selective NI, with least toxicity and drug like ADMET properties. Electronic, Steric requirements were defined using kohnone nearest neighbour- molecular field analysis (kNN-MFA) model of 3D-QSAR studies. Results generated by QSAR studies showed that model has good internal as well as external predictivity. Such defined requirements were used to generate new chemical entities which exhibit higher promising predicted activities. To check selective binding of designed NCE’s docking studies were carried out using the crystal structure of the neuraminidase enzyme having co-crystallized ligand Oseltamivir. Thus, molecular modelling provided a good platform to optimize the acyl thiourea pharmacophore for designing its derivatives having potent anti-viral activity*.*

## Introduction

Influenza virus infection is commonly known as flu and it is the contagious etiologic agent that causes an acute respiratory infection; hence it has always been a major threat (1) to human health worldwide and cause for economic burden. Currently, effective chemotherapy for influenza virus is also limited due to newly discovered drug resistance in mutant strain (2). Although the viral replicative cycle has revealed several potential molecular targets (M2 proteins endonuclease, hemagglutin and neuraminidase) that can be used as anti-flu for drug design (3-6). There are currently only a few licensed drugs available for influenza treatment. M2 inhibitors such as Amantadine and Rimantadine, which act specifically against Influenza A virus by blocking the ion channel of the M2 protein, provide only limited protection due to a narrow spectrum of activity (7). Neuraminidase (NA), also called sialidase, is the major surface glycoprotein that possesses enzymatic activity essential for viral replication and infection (8). The crystal structure of influenza virus Neuraminidase is well known and it is a critical enzyme in the infestation, replication, maturity and delivery of influenza virus (9). 

Though Neuraminidase inhibitors like Zanamivir displays excellent anti-viral activity when administered intranasal, it is less effective when delivered systemically. It has very low oral bioavailability and is rapidly eliminated by renal excretion (10).Oseltamivir is orally active, but it has been reported to cause vomiting, nausea and several allergic reactions. While remaining Neuraminidase inhibitors such as Peramivir, Laninamivir are still in phase III clinical trials. Peramivir shows less oral bioavailability than Oseltamivir. So, there is still an enormous need to design and identify new agents for the chemotherapy of influenza virus infection and formulate effective drugs for systemic administration. Therefore in present study we put forth following objectives:

1. To search the structural fragments responsible for selective NA inhibition.
2. To quantify the contribution of each structural fragments towards biological activity by comparing.
3. The contributing physicochemical parameters of required chemical groups in the given series.

The understanding of the enzyme’s structure and functions can be helpful for the development of novel Neuraminidase Inhibitors. Quantitative Structure Activity Relationships (QSAR) for different sets of compounds has been reported by Verma and Hansch (11) which gives seventeen different QSAR equations to understand chemical and biological interactions governing their activities toward influenza neuraminidase. Another QSAR model was developed with spatial, topological, electronic, thermodynamic and E-state indices on 30 thiourea analogues as influenza neuraminidase inhibitors using SYBYL 7.1 molecular modelling software and genetic algorithm method (12).

In this study, we performed 3D-QSAR using kNN-MFA method with the help of V-Life MDS 3.5. Considering electronic, steric parameters stated by 3D-QSAR studies, and also considering structural necessities for selective neuraminidase inhibitors at active binding sites of neuraminidase, the pharmacophore of acyl thiourea was optimized so as to inhibit neuraminidase efficiently. Optimized pharmacophore was used and little bit modified to generate basic templates so as to design new chemical entities using Combi-Lib tool of V-Life MDS 3.5. Generated library contains more than 100 entities, designed on the basis of equation of multiple linear regression studies. Out of 100, best 10 molecules were selected on the basis of predicted activity. Those best 10 molecules were subjected to docking studies. Docking studies were carried out by Glide tool of Schrodinger Inc. Software. QSAR (13-17) model gave good insights for developing new analogues as influenza virus NAI. On the basis of docking studies, it is concluded that designed entities show best binding interactions including Hydrogen bonds as well as Van-der-Waals forces within neuraminidase active binding site so as to inhibit neuraminidase efficiently for the treatment of influenza.

## Experimental

*Dataset*

A series (18) of total 28 compounds for whose absolute IC_50_ values is reported for their anti-influenza activity was used for QSAR studies (Table 1 and Table 2). Biological activity was expressed in terms of pIC_50 _which was calculated as log1/IC_50 _against neuraminidase enzyme. All QSAR models were generated using a Training set of 22 molecules. Test set of 6 molecules with varied chemical and biological activities were used to access the predictive power of generated QSAR models using training set of molecules. The sphere exclusion method was used for the selection of molecules in training and test sets.

Uni-Column statistics for training set and test set were generated to check correctness of selection criteria for trainings and test set molecules (Table 1).Selection of molecules in the training set and test is a key and important feature of any QSAR model, therefore due care was taken in such a way that biological activities of all compounds in test set lie within the maximum and minimum value range of biological activities of training set of compounds.

**Table 1:** Uni-Column statistics for Training and Test set of Compounds

**Training** **Set** **Average**	**Test** **Set** **Average**	**Training** **Set** **Max ** ^a^	**Test** **Set** **Max ** ^a^	**Training Set** **Min** ^a^	**Test Set** **Min** ^a^
-0.17912	-0.10595	1.09691	0.4437	-1.2672	-0.8567

a Higher the value of pIC50 (Greater +ve-value), higher is the potency.

**Figure 1:**
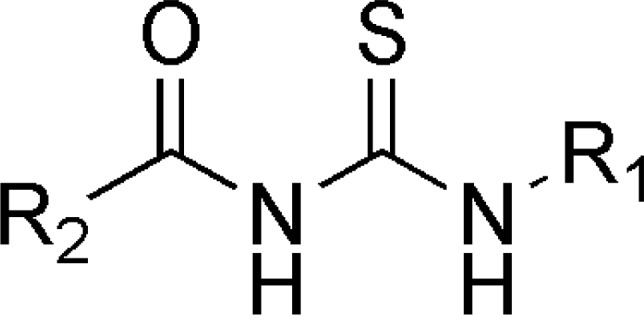
Acyl thiourea template

The common pharmacophore used for QSAR study is Acyl thiourea with R_1_ and R_2 _substitutions is represented (Figure 1). 

Selected series of acyl thiourea compounds with biological activity as neuraminidase inhibitors is depicted in tabular form along with training set and test set molecules (Table 2). Selected series is a collection of structures having wide range of neuraminidase inhibitory activity ranging from minimum 0.08 to maximum 18.5 µM.Validation of QSAR studies: Models generated by 3D-QSAR study was cross-validated using standard LOO procedure (19). 

*QSAR Studies*

*3D-QSAR Studies*

3D-QSAR studies were performed using V-Life Molecular Design Suite Software Version 3.5 (20). 3D-QSAR Studies were carried out by k Nearest Neighbor Molecular Field Analysis (kNN MFA) using Simulated Annealing (SA) variable selection method majority of its k-Nearest Neighbors in the training set (21-22).

**Figure 2:**
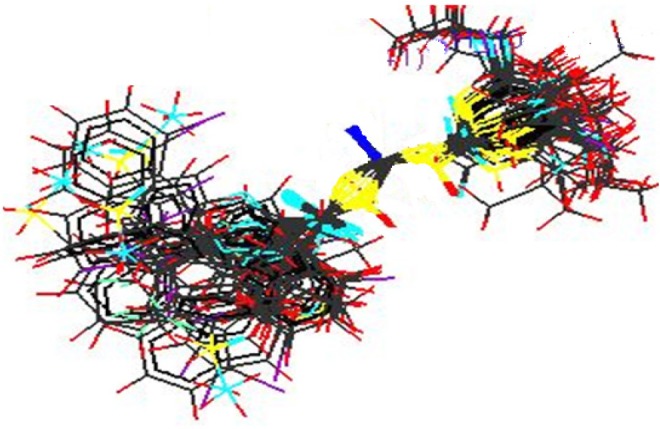
Alignment of Substituted Acyl Thiourea Derivatives using template based alignment method.

3D model was generated using following steps:

1. Molecules were optimized MMFF energy minimization method before alignment. Optimization is necessary process for proper alignment of molecules around template.

2. kNN-MFA method requires suitable alignment of given set of molecules; alignment was carried out by template based alignment method. Alignment of Substituted Acyl Thiourea Derivatives using template based alignment method is represented below (Figure 2).

1. This was followed by generation of common rectangular grid around the molecules, the steric and electrostatic interaction energies were computed at the lattice points of the grid using a methyl probe of charge +1.
2. The optimal training and test set were generated.
3. Models were generated by SA kNN MFA method.
4. The activity of the test set of compounds was predicted.
5. The 3D-QSAR model was evaluated using important statistical measures such as, ‘n’ is number of molecules (> 20 molecules), ‘k’ is number of descriptors in a model (statistically n/5 descriptors in a model), ‘q^2’^’ is cross-validated r^2^ (>.0.5), ‘pred_r^2^’ is r^2^ for external test set (>.0.5), ‘q^2^_Se’ is Standard Error in cross validation and ‘pred_r^2^_se’ is Standard Error in external validation.

*Optimization of Pharmacophore*

The information obtained from 3D QSAR study and active binding site was used to optimize the electrostatic and steric requirements as well as essential structural requirements for neuraminidase inhibition around the Acyl Thiourea nucleus for selective inhibition of Neuraminidase and in turn to enhance anti-influenza activity.

**Figure 3:**
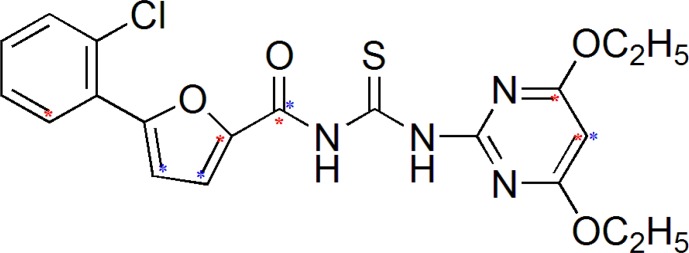
Most potent Compound from selected series

Generated 3D descriptors are correlated structurally and biologically with most potent compound (Figure 3) from the selected series of acyl thiourea having neuraminidase inhibitory activity up to 0.08 µM.

Considering above requirements obtained from QSAR studies and binding site studies, pharmacophore was optimized.

*Design of new chemical entities using designing of new templates and generation of library*

Information obtanined from QSAR studies and active binding site have helped us a lot to design new chemical Entities (NCE‘s).We have generated two templates using Lead Grow tool of V-Life MDS software and on the basis of these templates,we have generated more than hundrded molecules using CombiLib tool of V-Life MDS software.

*Molecular Docking studies*

Molecular docking studies were performed using Glide (5.0) (26) tool of Schrodinger molecular docking software. Those who had predicted activity as similar as pIC50 of most active molecule present in the reported series were selected for docking studies.

*ADMET Prediction*

All designed compounds were filtered by predicting their Absorption, Distribution, Metabolism, Excretion and Toxicity (ADMET) properties by means of Qikprop 2.2 Tool of, Schrodinger (Table 8).

## Results and Discussion

*Result of 3D-QSAR*

Series of Acyl thiourea derivatives was carefully divided into training set and test set. 3D-QSAR Studies were carried out by k Nearest Neighbor Molecular Field Analysis (kNN MFA) on the selective training set and test set as depicted in Table 2.

**Table 2:** Training set and Test Set from selected series of compounds for QSAR study with Acyl Thiourea pharmacophore

1) Training set molecules				
**Sr.** **No**	**R1**	**R2**	**IC50**	**pIC50**
1	4-Ethoxy-6-Methyl pyrimidine	5-(2-chlorophenyl)-2-furyl	1.65	-0.21748
2	4,6-diethoxy pyrimidine	5-(2-chlorophenyl)-2-furyl	0.08	1.09691
3	4-Hydroxy-6-Methyl pyrimidine	5-(2-chlorophenyl)-2-furyl	0.32	0.49485
4	4,6-dimethoxy pyrimidine	5-(2-chlorophenyl)-2-furyl	1.77	-0.24797
5	4,6-dichloro pyrimidine	5-(2-chlorophenyl)-2-furyl	14.5	-1.16137
6	4,6-dichloro pyrimidine	5-(4-nitrophenyl)-2-furyl	1.66	-0.22011
7	t-butyl amino carbonyl	5-(4-nitrophenyl)-2-furyl	1.30	-0.11394
8	t-butyl amino carbonyl	Phenyl	1.79	-0.25285
9	t-butyl amino carbonyl	Methyl	1.83	-0.26245
10	t-butyl amino carbonyl	(2,4-Dichloro-Phenyl)-OCH_2_	1.67	-0.22272
11	t-butyl amino carbonyl	2,6-Difluoro-Phenyl	1.43	-0.15534
12	t-butyl amino carbonyl	2-Methyl-1-(4-Chloro-Phenyl)-Propane	1.35	-0.13033
13	t-butyl amino carbonyl	3-(2, 2-dichloro ethenyl)-2,2-dimethyl cyclopropyl.	0.26	0.58503
14	4-Methoxy-6-chloro pyrimidine	5-(2-chlorophenyl)-2-furyl	1.29	-0.11059
15	4-Methyl-6-Hydroxy pyrimidine	6-Chloro-3-Pyridine	8.58	-0.93349
16	4,6-dimethoxy pyrimidine	5,6-Dichloro-3- Pyridine	18.5	-1.26717
17	4,6-dimethyl pyrimidine	Phenyl	2.1	-0.32222
18	4,6-diethoxy pyrimidine	2-Methyl-1-(4-Chloro-Phenyl)-Propane	0.31	0.50864
19	4,6-dimethoxy pyrimidine	3-(2-chloro-3,3,3-trifluropropenyl)-2,2-dimethyl cyclopropyl	0.97	0.01323
20	4,6-dimethyl pyrimidine	3-(2-chloro-3,3,3-trifluropropenyl)-2,2-dimethyl cyclopropyl	0.58	0.23657
21	4,6-dimethyl pyrimidine	2-Fluoro-4-Chloro-Phenyl	1.36	-0.133539
22	4-Methoxy-6-chloro pyrimidine	2-Fluoro-4-Chloro-Phenyl	5.1	-0.70757
2) Test set molecules.				
1	4-Hydroxy-6-Methyl pyrimidine	5-(4-nitrophenyl)-2-furyl	0.36	0.4437
2	t-butyl amino carbonyl	5-(2-Chloro-Phenyl)-2-Furyl	1.42	-0.152288
3	t-butyl amino carbonyl	3-(2-chloro-3,3,3-trifluropropenyl)-2,2-dimethyl cyclopropyl	0.51	0.29243
4	4-Methoxy-6-methyl pyrimidine	5-(4-nitrophenyl)-2-furyl	1.22	-0.08636
5	4,6-dimethyl pyrimidine	2-Chloro-3- Pyridine	7.19	-0.856729
6	4,6-dimethoxy pyrimidine	(2,4-Dichlorophenyl)-OCH_2_	1.89	-0.276462

**Table 3:** Statistical results of 3D QSAR generated by SA kNN MFA method for Acyl Thiourea Derivatives

**q2**	**pred_-r2**	**q2- se**	**K Nearest Neighbour**	**Pred- r2se**
**0.6840**	0.7697	0.2811	2	0.2972
**Contributing** **Steric** **Parameters**	S-333(-0.0053,0.0041)			
**Contributing** **Electronic** **Parameters**	E-449(0.0749,0.0796) E-450(-0.0456,0.0118)			
**Contributing** **Hydrophobic** **Parameters**	H-1047(0.3218,0.3622)			
**N**	24			

kNN MFA is a novel method which utilizes k-Nearest Neighbor (kNN) principle to correlate molecular field descriptors with biological activity. The kNN methodology relies upon a simple distance learning approach. In this method an unknown member is classified according to the majority of its k-Nearest Neighbors in the training set. The nearness is measured by an appropriate distance metrics (*e.g*., a molecular similarity measure calculated using field interactions of molecular structures). The standard kNN Method is implemented simply as follows:

1. The distances between an unknown object (u) and all other objects in the training set were calculated.
2. The k objects were selected from the training set most similar to object u, according to the calculated distances.

3. The object u was classified with the group to which the majority of the k objects belong. An optimal k-value is selected by optimization through the classification of a test set of samples or by Leave-One Out (LOO) cross validation (19). The variables and optimal k-values were chosen using different variable selection methods. Here we have used simulated annealing as variable selection method.

Statistical result of 3D-QSAR kNN MFA method is tabulated below (Table 3).

**Figure 4:**
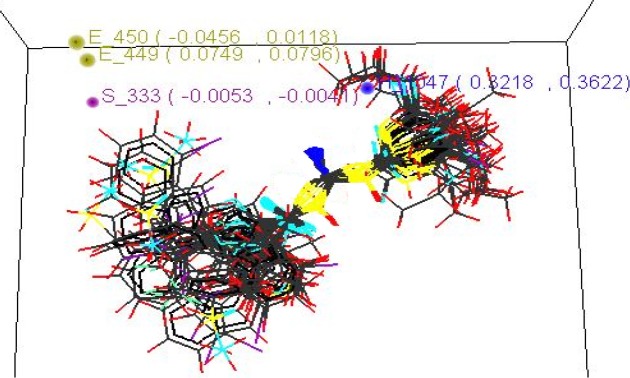
Stereo view of the molecular rectangular field grid generated around the superposed molecular units of Acyl Thiourea series using SA kNN-MFA Model

**Table 4:** Structures of designed compounds on the basis of template B with their predicted activity.

**Mol** **No.**	**R1**	**R2**	**Predicted activity**	**Lipinski’s score** **and** **Screen**
R-1	4-nitrophenyl	4-methoxyphenyl	0.8564	ADRXWS (6)
R-2	4-nitrophenyl	4-nitrophenyl	-0.3876	ADRXWS (6)
R-3	Benzyl	4-hydroxyphenyl	0.9765	ADRXWS (6)
R-4	3-aminophenyl	4-nitrophenyl	0.8450	ADRXWS (6)

**Figure 5:**
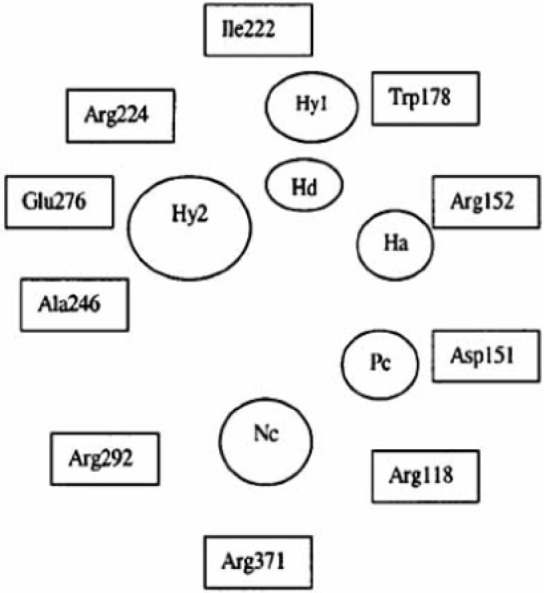
Stereo view of the molecular rectangular field grid generated around the superposed molecular units of Acyl Thiourea series using SA kNN-MFA Model

Model showed best internal as well as external predictivity as q^2^ = 0.6840, pred_r^2^ = 0.7697 and also error occurred during internal and external validation obtained was very low as q^2^_se = 0.2811, pred_r^2^_se = 0.2972.Stereo view of superposed molecular units of Acyl Thiourea series shows the generated data points around the pharmacophore in three dimensional manners, which is shown below (Figure 5).

Thus, SA KNN-MFA model leads to identification of various local interacting molecular features responsible for activity variation and hence provides direction for design of new molecules in a convenient way.

*Interpretation from 3D QSAR Studies and Correlation of 3D descriptors*

*Electronic Parameters*

3D QSAR studies revealed the electronic requirements around the acyl thiourea pharmacophore. The points those were found in SA KNN-MFA model are E_449, E_450 implying that these points are indeed significant for structure activity relationship and require the electronic properties as mentioned in the ranges in parenthesis for maximum biological activity.

Range for electronic descriptor E_450 (-0.0456, 0.0118).Descriptor ranging from negative to positive values specifies that the pharmacophore should be substituted with electronegative and electropositive groups so that it should increase the neuraminidase inhibiting activity, such as –OH, –Cl, -NO_2_, -OR and most potent compound possesses two electronegative groups such as –Cl, -OC_2_H_5_ from the parent series.

*Steric Parameters*

The steric points that were found in SA KNN-MFA model is S_333 implying that these points are indeed significant for structure activity relationship and require the steric properties as mentioned in the ranges in parenthesis for maximum biological activity. Range for steric descriptor is S_333 (-0.0053, -0.0041). Descriptor showing negative values specifies that the pharmacophore should be substituted with sterically less bulky group so that it can increase the NA inhibiting activity, such as lower alkyl groups, unsubstituted or mono substituted aryl rings. 

*Study of Structural Necessity for Neuraminidase Inhibition*

**Figure 6:**
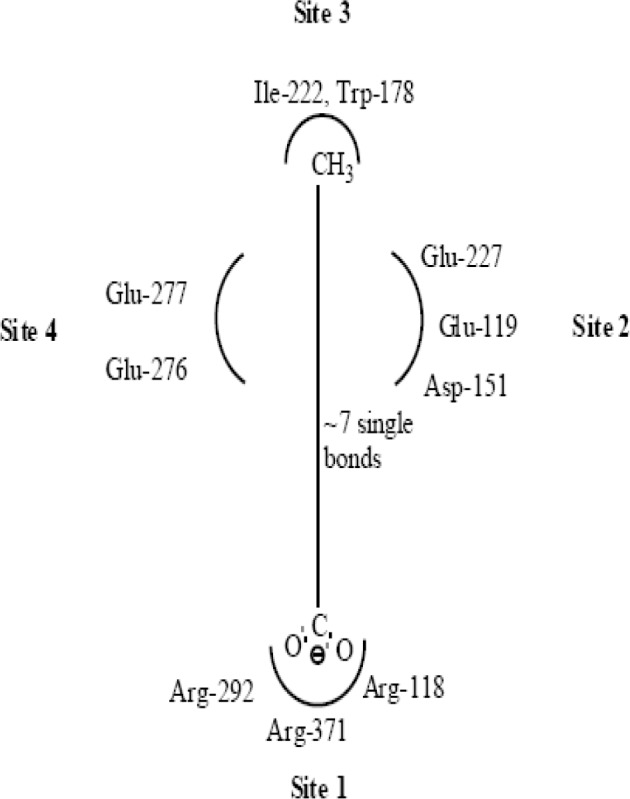
Important structural features for potent inhibitor and corresponding neuraminidase residues (23)

Wang and Wade have reported guidelines for structural modification of developing neuraminidase inhibitors and the design of novel inhibitors in order to optimize inhibitory activity (Figure 6) (23). Since the catalytic site of influenza virus Neuraminidase was totally conserved among all influenza viral strains, an ideal drug which effectively blocked one neuraminidase would be effective at blocking all other neuraminidases, even those on viruses which have not yet appeared in humans (8). 

Thus, along with QSAR study, we are herein considering the essential structural necessities which should be present in the acyl thiourea pharmacophore. It will be beneficial in such a way that we can design better compounds which would fit into neuraminidase binding site perfectly and would show best interactions than standard, marketed neuraminidase inhibitors. 

A negatively charged (Nc) group that makes strong charge-charge interactions with the triarginyl pocket as Arg 292, Arg 371 and Arg 118 is highly favorable for binding. Positively charged (Pc) group is favoured for binding by the electrostatic interactions with residue Asp151.While, Arg 152 requires hydrogen bond acceptor (Ha) group for binding. Trp 178 requires such a structural functionality which should be hydrogen bond donor (Hd) as well as small hydrophobic group for favourable binding. While, large hydrophobic group contributing to the van der Waals interaction should be preferred for three different amino acid residues such as Arg 224, Ala 246 and Glu 276.

**Figure 7:**
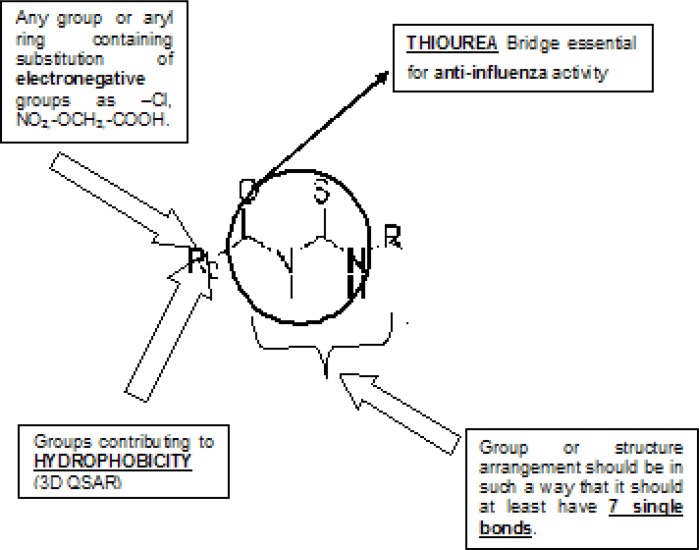
Diagram of neuraminidase sites S1-S4 and important nearby residues (25).

Along with structural necessities for neuraminidase inhibition, we are considering air plane model of neuraminidase enzyme, so as to design potential derivatives with excellent anti-influenza activity (24). Wang and co-workers (24) derived an airplane model of the neuraminidase active site as illustrated in (Figure 7) to summarize the basic structural requirements of potent neuraminidase inhibitors. The active site of neuraminidase has four main well conserved binding sites. The centre of site 2 is about 6 A.U. from site 1 and about 4 A.U. from site 3, while site 4 is about 6 A.U. from site 1 and 5 A.U. from site 3. Sites 1 and 3 are separated by 9-10 A.U. or about 7 single bond lengths.

Gong and co-workers have proposed about the pocket study of neuraminidase active site (8). 

By taken into consideration, structural necessity and airplane model and pockets of neuraminidase enzyme, we will design such new chemical entities which are much potential than standard and selective inhibitors.

*Design of New Chemical Entities*

**Figure 8:**
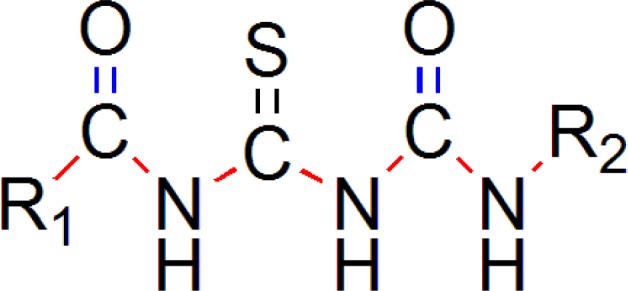
Pharmacophore Requirement of Acyl thiourea Derivatives Generated for Selective Inhibition of neuraminidase for anti-influenza activity

Designed compounds generated in this way were then screened by three type of screening methods; Lipinski’s rule and prediction of activity using multiple linear regression equation [pIC_50_ = 0.250765 T_C_N_6 + 0.106694 T_C_O_2 + 0.637398 MMFF_2 - 0.623115 chiV3 + 6.1896] obtained by the two dimensional QSAR studies and to ensure drug like pharmacokinetic profile by prediction of ADMET properties for finding a new compounds having neuraminidase inhibitor and anti-influenza activity (20). New chemical entities were generated using optimized pharmacophore shown in (Figure 8).

More than one hundreds of molecules were generated using CombiLib tool which follows the Lipinski’s rule, but we have selected only 10 most active molecules on the basis of predicted activity. The most potent compounds have positive values of pIC_50_, similarly the predicted activity values for new chemical entities were found to lye towards positive side.Compounds qualifying all required parameters set for Lipinski’s Screen/filter are indicated by ADRXWS strings. The columns containing the Lipinski’s screen score and other column containing the strings of alphabets, ADRXWS indicate that all 6 conditions are satisfied by that corresponding compound. Lesser the screen score, lesser is the pharmacokinetic compatibility (drug likeliness) for that designed compound.

*Generation of Templates*

We have generated two templates considering above stated pharmacophore requirements. Designed templates consist of dicarbonyl groups which are different than parent series, while parent series has single acyl group in it, we have placed another acyl group instead of any aryl ring containing oxygen atom, as in parent series has furan ring in it (19). 

**Figure 9:**
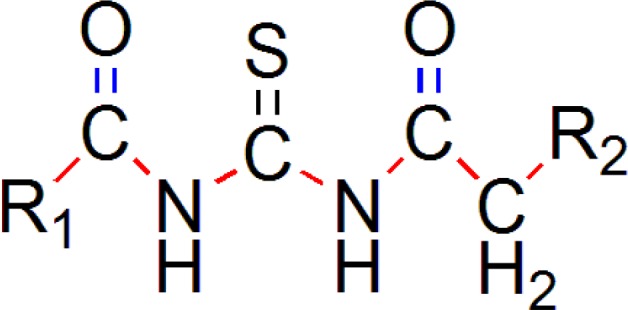
Template A

**Figure 10:**
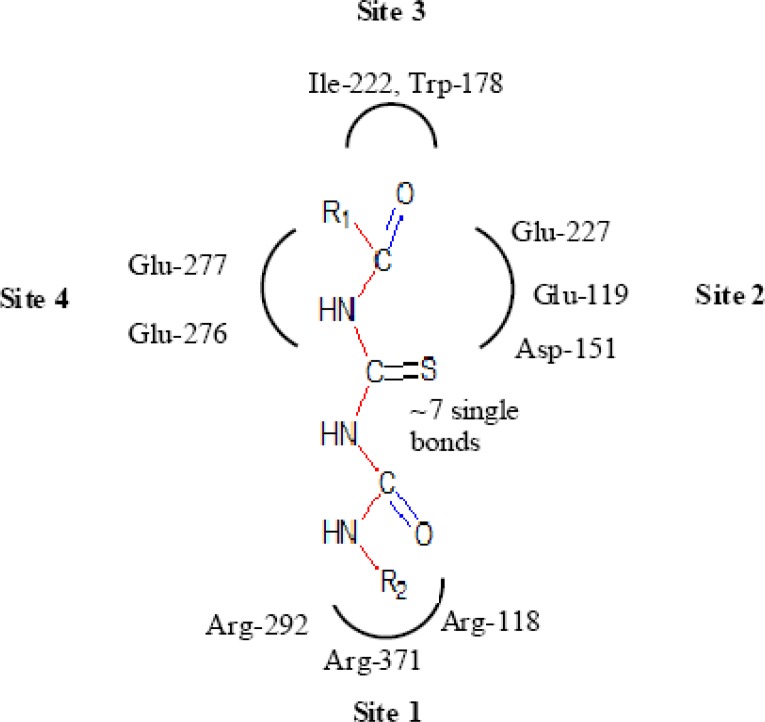
Template B

One template (Figure 9) consists of 3 –NH groups in the pharmacophore, while another template (Figure 10) consists of 2 –NH groups, one –NH group is isosterically replaced by –CH_2_ group.

**Figure 11:**
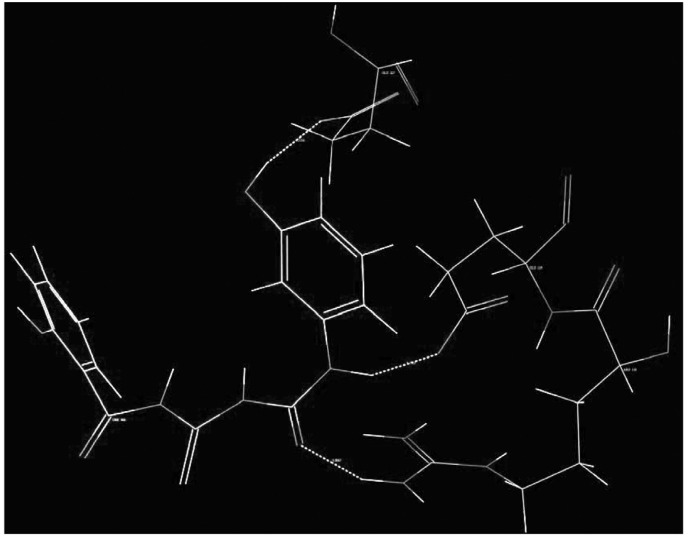
Designed template with active binding site

Both of the templates have seven single bonds between R^1 ^and R^2^ substituents as it is basic structural requirement for neuraminidase inhibition, where in case of R^1^ and R^2^, we have used aryl ring with electronegative and electropositive groups such as–OH,-COOH,-NO_2_ and –OCH_3, _–NH_2 _and aryl ring also contributes to hydrophobicity, as correlated in 3D-QSAR studies. Those seven single bonds are represented in red colour in designed template (Figure 11). Even placement of extra carbonyl functionality and aryl ring with electronegative carboxyl group in the acyl thiourea pharmacophore becomes helpful for binding it with triarginyl residues as discussed above (Figure 6,7).

*Generation of Combinatorial Library*

We have generated more than hundrded molecules using CombiLib tool (20) which follows the Lipinski’s rule, but we have selected only 10 most active molecules on the basis of their predicted activity using Multiple Linear regression equation.

We have selected only four molecules from the B template (Table 4) considering promising predicted activity.While from A template we have selected seven molecules (Table 5) on the basis of predicted activity.

**Table 5:** Structures of designed compounds on the basis of template A with their predicted activity.

**Molecule** **No.**	**R1**	**R2**	**Predicted activity**	**Lipinski’s score and Screen**
R-5	3-nitro phenyl	3-carboxy phenyl	-0.03206	ADRXWS (6)
R-6	2-hydroxy phenyl	3-carboxy phenyl	-0.06934	ADRXWS (6)
R-7	2-hydroxy phenyl	3-hydoxy phenyl	-0.156	ADRXWS (6)
R-8	4-nitrophenyl	3-carboxy phenyl	-0.18876	ADRXWS (6)
R-9	Benzyl	3-hydoxy phenyl	-0.38875 -0.17141	ADRXWS (6)
R-10	4-methoxy phenyl	3-carboxy phenyl	-0.39318	ADRXWS (6)

Out of total 11 designed new chemical entities only 3 compounds viz. R-8,R-9 and R-10 show predicted activity less potent as compared with most potent compound from the parent series with pIC50 1.09691,because higher the value of pIC50 more potent is the entity.

**Table 6:** Results of Molecular Docking Studies performed using extra precision mode of Glide (34).

**Molecule** **No.**	**G-** **Score**	**E-** **model**	**No of** **H** ^ a^ **Bond**	**No. of** **Good** **Vdw** ^ b^	**No. ofBad** **Vdw** ^ b^	**No. ofUgly** **Vdw** ^ b^
R-7	-5.98	-51.6	3	220	12	3
R-8	-5.58	-50.5	4	233	10	3
Peramivir	-5.53	-45.5	7	278	14	2
R-4	-5.14	-47.3	2	241	7	1
R-10	-5.07	-43.2	8	150	9	2
R-9	-4.83	-47.9	2	210	9	2
R-2	-4.79	-41.2	3	234	11	1
R-5	-4.58	-47..3	3	134	6	1
Oseltamivir	-4.57	-49.3	4	266	8	1
R-3	-4.27	-51.3	2	233	6	0
R-6	-3.91	-58.0	6	167	7	0
R-1	-2.74	-2.74	2	173	6	0

^a^Hydrogen Bond.

^b^Van der Waals forces.

*Molecular Docking studies*

Molecular Docking is a key tool in structural molecular biology and computer-assisted drug design. Selected most active molecules were docked on crystallographic structure of neuraminidase enzyme available in the RCSB PDB Database (Code: 3b7e) co-crystallized with the ligand Zanamivir (27). A molecular docking study helps to determine possible interaction of new chemical entities with the enzyme on PDB (3b7e).

The goal of ligand–protein docking is to predict the predominant binding mode(s) of a ligand with a protein of known three-dimensional structure. Virtual screening on the basis of molecular descriptors and physicochemical properties of active ligands has great usefulness in finding hits and leads through library enrichment for screening (28), a strategy that is also well-used for reducing and enriching the library of ligands for molecular docking; there are recent reports that ligand shape-matching does as well as, if not better than, docking (29). 

Glide was found to produce least number of inaccurate poses and 85% of Glides binding models had an RMSD of 1.4 A^0^ or less from native co-crystallized structures (30). The Glide docking program approximated a complete systematic search of the conformational, orientation and positional space of the docked ligand molecules in to the receptor (protein) binding pocket. 

*Overview of docking of New Chemical Entities*

All the designed compounds that show good predicted activity and follows Lipinski’s rule were docked into neuraminidase enzyme (pdb code: 3b7e) to study the binding mode of designed compounds. Further screening to sort out the best compound having good binding affinity which was compared with binding mode of Standard Neuraminidase Inhibitors, *e.g*. Oseltamivir, Zanamivir and Peramivir results of which are depicted in (Table 6 and 7).

The reliability of the docking results was first checked by comparing the best docking poses obtained for the co-crystallized inhibitor with its bound conformation. 

As a result, a root mean square deviation (RMSD) of 0.7 Å was found suggesting that the docking procedure could be relied on to predict the binding mode of our compounds. 

**Figure 12:**
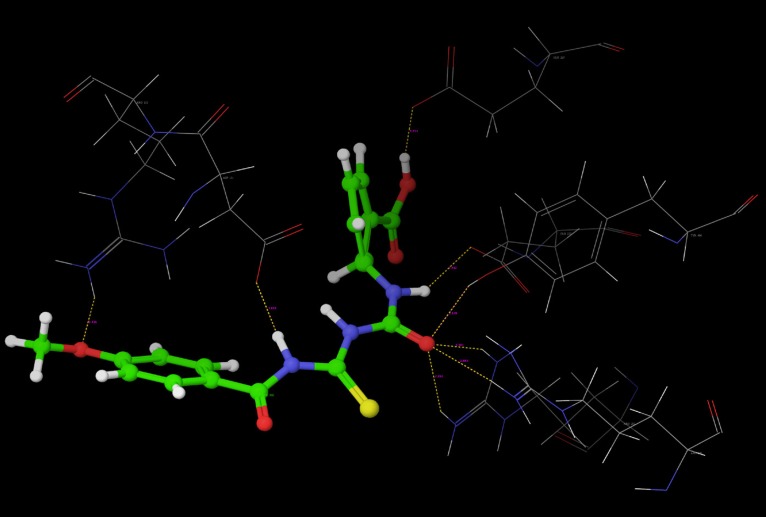
Hydrogen bond interaction of R-7

**Figure 13:**
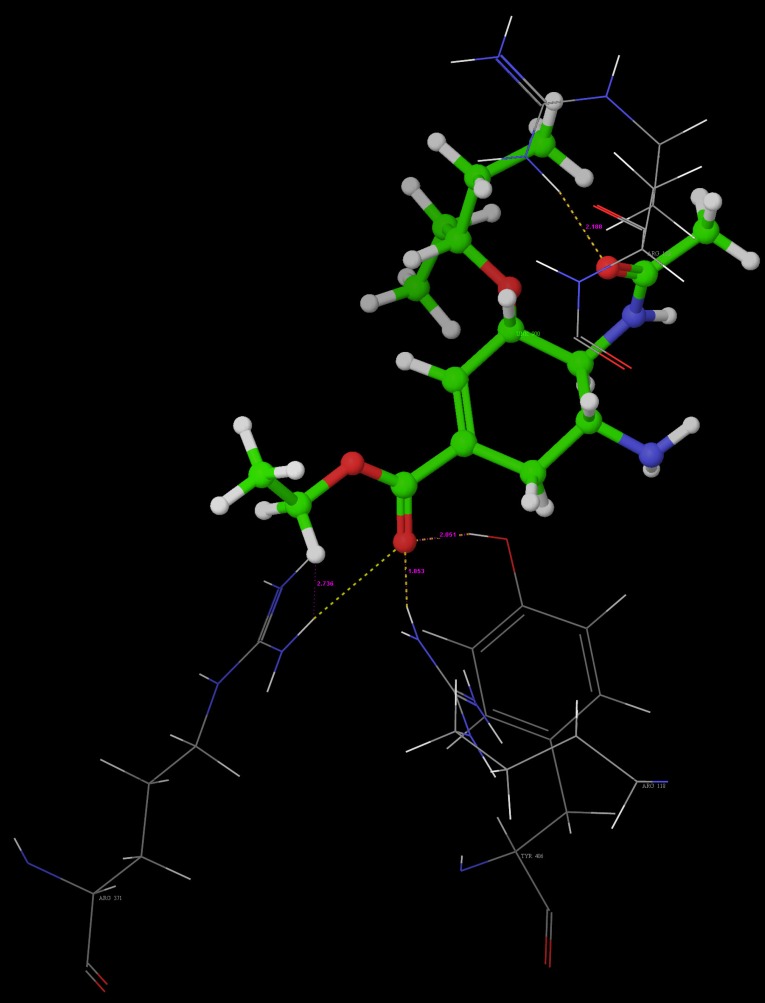
Hydrogen bond interaction of R-10

**Figure 14:**
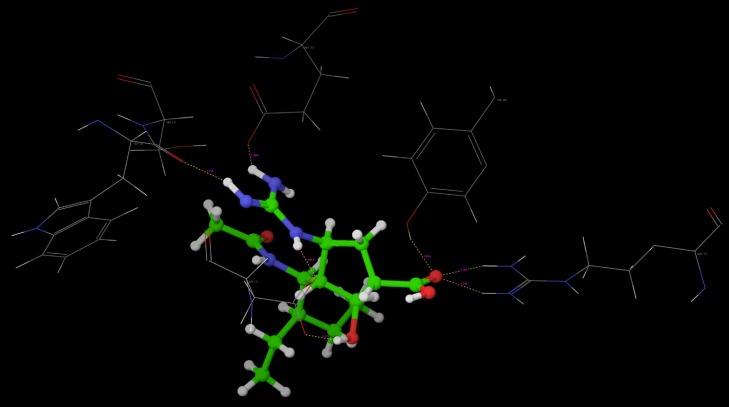
Hydrogen bond interaction of Oseltamivir

**Figure 15:**
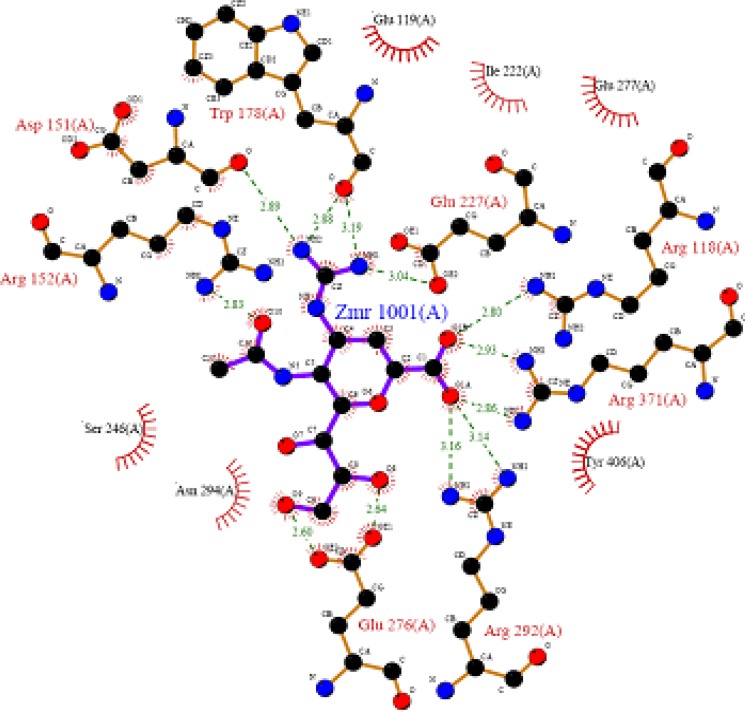
Hydrogen bond interaction of Peramivir

Poses of interactions of ligands along with standard are represented in (Figure 12, 13, 14 and 15).

*Key findings of overall Docking studies*

The close inspection of result of molecular docking studies indicated that the designed compounds docked better than ligand Oseltamivir and Peramivir. Following are the reasons for the same.

*1. G-Score*

Molecules R-7 and R-8 show highest G-score than both the standards Peramivir and oseltamivir. While, molecules such as R-2, R-4, R-5, R-9 and R-10 show higher G-score than oseltamivir.

*2. Hydrogen bond interactions*

Molecule R-10 shows highest hydrogen bond interactions *i.e*. 8 than Peramivir and Oseltamivir, which show 7 and 4 interactions respectively. While, R-6 shows 6 hydrogen bond interactions which are more than oseltamivir.

*3. Van der Waals interactions*

R-1, R-3, R-4, R-5, R-6 show less bad van der Waals interactions than oseltamivir and Peramivir, where only Peramivir shows highest bad van der Waals interactions as 14 and oseltamivir shows 8 bad van der Waals interactions. Molecules R-1, R-3 and R-6 show zero ugly forces, where oseltamivir and Peramivir show 2 and 1, respectively.

4. In all the bad and ugly contacts penalize the G-score and overall conformation of ligand does matter a lot while binding to amino acid residues at active binding site. This also penalizes the energy of the model.

Key interactions are depicted in tabular form (Table 7). It was observed from docking studies that all ligands lie in the same pocket of neuraminidase binding site of enzyme containing Arg 152, Tyr 406, Arg 371, Arg 118, Glu 227, Glu 277, Asp 151, Arg 292, Asn 294 and Ser 246 amino acids. Thus designed compounds showed a good binding affinity for interaction with neuraminidase active binding site.R-7, R-8, R-9 and R-10 show Hydrogen bond interaction with different arginine residues, in this case one carbonyl group, a negatively charged group from dicarbonyl thiourea interacts with positively charged groups or ions such as –NH_2_ or H^+^ of OH arginine residues. Here, one of structural necessity for selective neuraminidase inhibition is satisfied.

R-7, R-8, R-9 and R-10 show electrostatic interaction with different glutamic acid residues. In their case, either a positively charged group such as –NH_2_ or positively charged ion H^+^ of –OH or –COOH interacts with carbonyl or carboxyl group of glutamic acid residues, satisfying structural necessity for selective neuraminidase inhibitor.

Oseltamivir shows Hydrogen bonding with amino acids Arg 152, Tyr 406, Arg 371, Arg 118 which are one of essential interactions for neuraminidase inhibition, which is described previously in study of structural necessity for neuraminidase inhibition. Distance of hydrogen bonds in NCEs and standard compounds also explain better interaction (Table 7) with neuraminidase binding site in 3b7e pdb.

The hydrogen bond distance in case of compound R-7 with Glu 227 (1.793 A.U.) which is less as compared to the hydrogen bonding interaction in case of Peramivir which is 2.477 A.U. Hence, less hydrogen bonding distance than standards is required for better binding which is reported in NCEs during docking study. 

G-score is calculated taking all above aspects in to consideration, while designing new chemical entities to inhibit Neuraminidase more effectively and in turn to optimize the pharmacophore required for selective inhibition of Neuraminidase.

**Table 7:** Key interactions with distances and involved groups

**Molecule No.**	**H-bond interaction** **With ** **amino acid**	**H-bond interaction** **in A.U.**	**Functional Group of ** **structure involved ** **in bonding**	**Amino acid Residue involved in bonding**
R-7	Glu 119 Arg 118 Glu 227	1.793 1.807 1.779	-NH -C=O H of OH	-COOH -NH2 -COOH
R-8	Glu 119 Arg 118 Glu 227 Tyr 406	1.747 1.913 1.738 1.929	-NH -C=O H of COOH -C=O	-COOH H of OH -COOH -NH2
Peramivir	Trp 178 Glu 227 Tyr 406 Asp 151(2) Arg 371(2)	1.960 2.477 1.985 a) 1.803 b) 2.315 a) 2.207 b) 1.966	-NH of guanidine -NH of guanidine -C=O of COCH3 a) -NH of guanidine b)H of OH a) -C=O of COCH3 b) -C=O of COCH3	-C=O H of OH H of OH a)-C=O b) -C=O -NH2 -NH2
R-4	Asp 151 Trp 178	2.068 1.976	-NH -NH	-C=O -C=O
R-10	Asp 151 Arg 292 Arg 371(2) Glu 277 Tyr 406 Glu 227 Arg 152	1.851 2.992 a)2.353 b)2.101 2.482 2.179 1.731 2.135	-NH -C=O a) -C=O b) -C=O -NH -C=O H of OH O of OCH3	-C=O -NH a)-NH b)-NH -C=O H of OH -C=O -NH
R-9	Glu 119 Arg 118	1.938 1.894	-NH -C=O	-C=O -NH
R-2	Asn 294 Ser 246 Asn 221	2.3 2.102 2.138	O of NO2 O of NO2 O of NO2	-NH -NH -NH
R-5	Asp 151 Tyr 406	2.213 2.412	-NH -C=O	-C=O H of OH
Oseltamivir	Arg 152 Tyr 406 Arg 371 Arg 118	2.18 2.051 2.470 1.853	-C=O of COCH3 -C=O of COCH3 -C=O of COOC2H5 -C=O of COOC2H5	-NH -OH -NH -NH

*ADMET Prediction*

Prediction of ADMET properties was used as last screen to sort out those compounds that already follow Lipinski’s rule and show good predicted activity and binding conformation at Neuraminidase receptor.

The parameters illustrated in (Table 8) Qikprop analysis show significant results. CNS parameter is related with absorption of entity through Blood brain barrier, standard limit for CNS is -2 to +2, where -2 shows inactive CNS penetration and +2 shows active CNS penetration. All the designed entities show satisfactory results, with negative values, indicating poor CNS penetration. % Oral Absorption parameter is related with extent of oral absorption of drug, indicating suitable route of administration, if it is going to be formulated. If entity shows more than 80% oral absorption, it is considered to be highly absorbed. While if any entity shows less than 25% oral absorption, it is considered to be poorly absorbed. Metabolites suggest the number of metabolites which will possibly generate after undergoing metabolic changes, number of metabolites should range from 1-8.

**Table 8:** Prediction of ADMET properties

**Molecule No.**	**Molecular** **weight**	**CNS**	**% Oral** **Absorption**	**No.of possible metabolites**
R-7	331.345	-2	78.416	3
R-8	388.354	-2	48.485	2
Peramivir	328.411	-2	29.001	3
R-4	358.371	-2	73.409	4
R-10	373.383	-2	73.351	2
R-9	329.373	-2	84.192	3
R-2	388.354	-2	68.372	3
R-5	358.371	-2	43.878	3
Oseltamivir	312.408	-1	68.391	3

## Conclusion

The thorough analysis of results of 3D QSAR studies and the structural necessities for neuraminidase inhibition have helped us to make a decision about the electronic, steric, hydrophobic nature of substitution pattern around the selected acyl thiourea pharmacophore. Two different Templates were generated on the basis of above information using lead grow tool of V-life Molecular design suite. Using the information, the New Chemical Entities (NCEs) were designed using CombiLib tool with the help of multiple linear regression equation and activities were also computed for the designed NCEs.

The major reason for failure of NCEs at latter stages of drug discovery process *i.e.* drug like pharmacokinetic profile set up using Qikprop (33) 2.2 Tool of, Schrodinger; so that only drug like NCEs would be generated and resultant NCEs would not have the pharmacokinetic inadequacies. The generated NCEs were analyzed by Lipinski’s screen. Results indicated that designed NCEs are satisfying all the parameters set for Lipinski’s screen. The most potent derivatives were subjected to molecular docking studies to get further insights of interactions of NCEs with neuraminidase.

In present study, we performed molecular modeling study demonstrating that acyl thiourea derivatives inhibit neuraminidase by binding to the site. The results of dry lab work will be analyzed thoroughly to find out correctness of the rational used for the design of NCEs in general and optimization of pharmacophore for selective neuraminidase inhibition of in particular.

## References

[1] Monto AS, Acuzio IA, LaMontague JR (1997). Pandemic Influenza confornting reemergent threat. Threat J.Infect. Dis..

[2] Hayden FG, Belshe RB, Glover RD (1989). Emergence and apparent transmission of rimantadine-resistant influenza A virus in families. N Engl. J. Med..

[3] Hay AJ, Wolstenholme AJ, Skehel JJ (1985). The molecular basis of the specific anti-influenza action of amantidine. EMBOJ.

[4] Hastings JC, Selnick HG, Wolanski BS, Tomassini JE (1996). Anti-influenza virus activities of 4-substituted 2, 4-dioxobutanoic acid inhibitors. Antimicrob Agents Chemother..

[5] Mammen M, Dahmann G, Whitesides GM (1995). Effective inhibitors of hemagglutination by influenza virus synthesized from polymers having active ester groups. Insight into mechanism of inhibition. J. Med. Chem..

[6] Colman PM, Krug RM (1989). The Influenza Viruses: Influenza Virus Neuraminidase Enzyme and Antigenin the influenza viruses..

[7] Erik De, Clercq J (2001). Anti-viral drugs: current state of the art. Clin Virol..

[8] Gong J, Xu W, Zhang J (2007). Structure and functions of influenza virus neuraminidase. Curr Med. Chem..

[9] Varghese JN, Laver WG, Colman PM (1983). Structure of the influenza virus glycoprotein antigen neuraminidase at 2.9 Å resolution. Nature.

[10] Ryan DM, Ticehurst J, Dempsey MH (1995). GG167 (4-guanidino-2, 4-dideoxy-2,3-dehydro-N-acetylneuraminic acid) is a potent inhibitor of influenza virus in ferrets. Antimicrob Agents Chemother.

[11] Verma RP, Hansch CA (2006). QSAR study on influenza neuraminidase inhibitors. Bioorg Med. Chem..

[12] Nair PC, Sobhia ME (2008). Quantitative structure activity relationship studies on thiourea analogues as influenza virus neuraminidase inhibitors. Eur J. Med. Chem.

[13] Golbraikh A, Tropsha A (2002). Beware of q2. J Mol. Graph. Model.

[14] Rogers D, Hopfinger AJ (1994). Application of genetic function approximation to quantitative structure-activity relationships and quantitative structure-property relationships. J Chem. Inf. Comput. Sci..

[15] Kubinyi H (1994). Variable selection in QSAR studies. I. An evolutionary algorithm. Quant. Struct-Act. Relat..

[16] So SS, Karplus M (1996). Evolutionary optimization in quantitative structure−activity relationship: an application of genetic neural networks. J Med. Chem..

[17] Kubinyi H, Hambrecht FA, Mietzner T (1998). Three-dimensional quantitative similarity-activity relationships (3d qsar) from seal similarity matrices. J Med. Chem..

[18] Sun C, Zhang X, Huanga H, Zhou P (2006). Synthesis and evaluation of a new series of substituted acyl(thio)urea and thiadiazolo [2,3-a] pyrimidine derivatives as potent inhibitors of influenza virus neuraminidase Bioorg. Med Chem..

[19] Ajmani S, Jadhav K, Kulkarni SA (2006). Three-dimensional QSAR using the k-nearest neighbor method and its interpretation. J Chem. Inf. Model..

[20] (2004). Molecular Design Suite version 3.5. VLifeMDS.

[21] Zheng W, Tropsha A (2000). Novel variable selection quantitative structure-property relationship approach based on the k-nearest-neighbor principle. Chem Inf. Comput. Sci..

[22] Ajmani S, Jadhav K, Kulkarni SJ (2006). Three-dimensional QSAR using the k-nearest neighbor method and its interpretation. Chem Inf. Comput. Sci..

[23] Wang T, Wade RC (2001). Comparative binding energy (COMBINE) analysis of influenza neuraminidase-inhibitor complexes. J Med. Chem..

[24] Wang GT, Chen Y, Wang S, Gentles R, Sowin T, Muchmore W, Kati S, Giranda V, Stewart K, Sham HL, Kempf D, Laver WG (2001). Design, synthesis, and structural analysis of influenza neuraminidase inhibitors containing pyrrolidine cores. J Med. Chem..

[25] Taylor NR, Von Itzstein M (1996). Molecular modelling studies on ligand binding to sialidase from influenza virus and the mechanism of catalysis. J Comput. Aided Mol. Des..

[26] Friesner RA, Murphy RB, Repasky MP, Frye LL, Greenwood JR, Halgren TA, Sanschagrin PC, Mainz DT (2006). Extra precision glide: docking and scoring incorporating a model of hydrophobic enclosure for protein-ligand complexes. J Med. Chem..

[27] Xu X, Zhu X, Dwek RA, Stevens J, Wilson IA (2008). Structural characterization of the 1918 influenza virus H1N1 neuraminidase. J Virol..

[28] Pozzan A (2006). Molecular descriptors and methods for ligand based virtual high throughput screening in drug discovery. Curr Pharm. Des..

[29] Hawkins PC, Skillman AG, Nicholls A (2007). Comparison of shape-matching and docking as virtual screening tools. J Med. Chem..

[30] Meclellan LM, Sokol LM, Kontoyiamn M (2004). Evaluation of docking performance: comparative data on docking algorithms. J Med. Chem..

